# Supplementary material for: A Global Screen for Assembly State Changes of the Mitotic Proteome by SEC-SWATH-MS
Source: Cell Syst. 2020 Feb 26;10(2):133–155.e6. doi: 10.1016/j.cels.2020.01.001 (PMC7042714; doi:10.1016/j.cels.2020.01.001)

Q9ULW0 | TPX2_HUMAN | TPX2 C20orf1 C20orf2 DIL2 HCA519
Monomer MW [kDa]: 85.653 Monomer expected elution fraction: 42

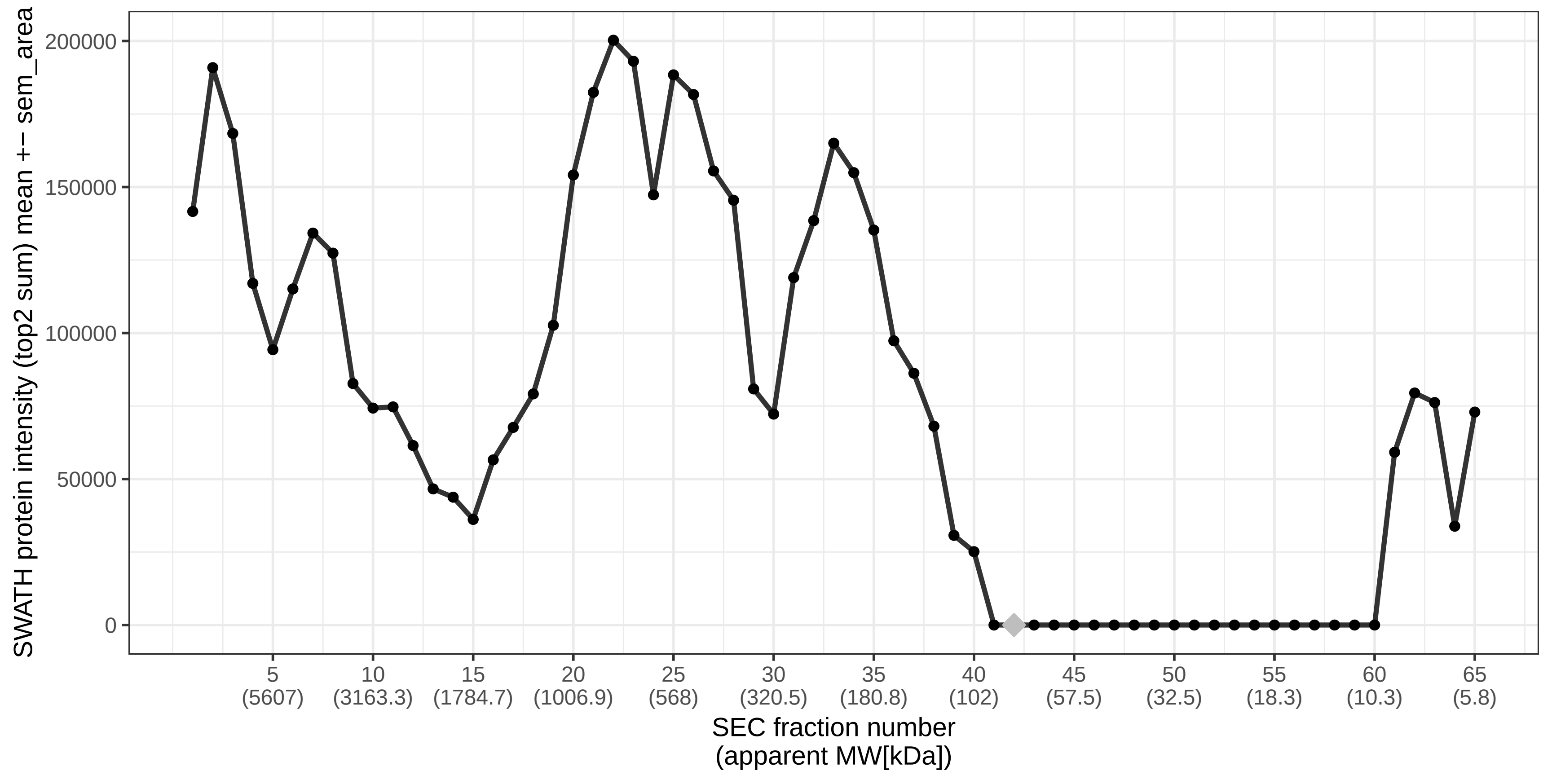

Supplement: Data S1. SEC-SWATH-MS Protein Chromatograms, Related to Figure 1 [file mmc6.zip › SECchrom_Q9ULW0_TPX2_HUMAN_TPX2_C20orf1_C20orf2_DIL2_HCA519.pdf]
